# Knowledge, attitudes, and practices of local land services staff regarding Japanese encephalitis virus: A one health perspective

**DOI:** 10.1016/j.onehlt.2026.101435

**Published:** 2026-05-03

**Authors:** Jennifer White, Nicole Schembri, Keeley Allen, Rebecca Forsythe, David N. Durrheim

**Affiliations:** a Health Protection, Hunter New England Local Health District, Booth Building, Wallsend Health Services Longworth Avenue, Wallsend, NSW 2287, Australia; b School of Medicine and Public Health, University of Newcastle, University Drive, Callaghan, NSW 2308, Australia; c One Health Unit, Health Security and Emergency Management Division, interim Australian Centre for Disease Control, Surry Hills NSW 2100, Australia; d Animal Biosecurity, NSW Department of Primary Industries and Regional Development, Orange NSW 2800, Australia; e The Peter Doherty Institute of Infection and Immunity, University of Melbourne, Elizabeth Street, Melbourne, VIC 3000, Australia

**Keywords:** Japanese encephalitis virus, Arbovirus, Culex, Occupational health, Vaccination, Vector-borne disease, Mixed methods, Australia

## Abstract

Japanese encephalitis virus (JEV) is a mosquito-borne zoonotic disease with a transmission cycle, involving *Culex* spp. mosquitoes, pigs, and waterbirds, highlighting the importance of a One Health approach to its prevention and control. This mixed-methods study assessed knowledge, attitudes, and practices among Local Land Services (LLS) staff in rural New South Wales (NSW), occupationally at considerable risk of JEV exposure, to inform integrated prevention strategies. An online questionnaire (*n* = 46) and a focus group (*n* = 20) explored their awareness, vaccination status, and personal protective practices (PPP) adopted. While most participants recognised JEV as a zoonotic disease, misconceptions about transmission and inconsistent use of repellents were commonplace. Only 18% were vaccinated, but vaccination rates were higher when the workplace facilitated access (OR = 12.4, 95%CI 1.28–581.7). Qualitative findings revealed that seasonal cues influenced PPP, that targeted practical communication was preferred, and that cross-sector collaboration between veterinary, agricultural, and public health agencies was ad hoc yet valued. Findings underscore the need for coordinated, workplace-based prevention programs embedded within Work Health and Safety frameworks, supported by consistent cross-sector collaboration and context-specific communication. Integrating efforts within a One Health framework is critical to strengthening preparedness against emerging zoonotic threats in agricultural settings.

## Highlights

• Study shows vaccination uptake strongly linked to workplace provision (OR 12.4), highlighting employer-facilitated access.
• We identify 3 themes—risk awareness, cues, and cross-sector collaboration—addressing barriers and preferred prompts.
• We recommend embedding Japanese encephalitis in One Health–aligned workplace-based prevention strategies, providing seasonal risk alerts, and coordinating multi-agency messaging to sustain protective behaviours.

## 1 Introduction

Japanese encephalitis (JE) is a serious mosquito-borne zoonosis caused by the Japanese encephalitis virus (JEV), transmitted mainly by *Culex* spp. mosquitoes breeding in freshwater environments (1). In Australia, ardeid waterbirds are considered important reservoir hosts for the JEV, maintaining the virus in natural transmission cycles and potentially contributing to its geographic spread through migratory movements [2], [3]. In contrast, feral pigs act as key amplifying hosts, while humans and horses as dead-end hosts [4], [5]. This transmission cycle exemplifies the interdependence of human, animal, and environmental systems central to the One Health framework (6). Climate change and environmental alterations have expanded mosquito habitats, facilitating JEV spread into new regions including urban and temperate zones [7], [8]. Although most JE infections in humans are asymptomatic, approximately 1% progress to neuroinvasive disease with significant morbidity and mortality (9).

In 2022, JEV emerged across southeastern Australia, with human, pig, and mosquito detections in southern Queensland (10), New South Wales (NSW) (2), Victoria (11), and South Australia (12). The outbreak, declared a Communicable Disease Incident of National Significance, marked the first widespread transmission on mainland Australia. By June 2025, 50 human cases and 11 deaths had been reported [13], [14], suggesting JEV has become endemic in southeastern Australia (15).

Vaccination is recommended for people living or working in high-risk areas, particularly those exposed to pigs, wetlands, or mosquitoes (16). Personal protective practices (PPP), such as the of DEET-based repellents including reapplication, protective clothing, and mosquito control, are considered essential for limiting infection risk (16). However, the effectiveness of such measures depends on awareness, accessibility, and workplace support (17).

Local Land Services (LLS) is a regional NSW Government agency responsible for agricultural extension, biosecurity and animal health management, and natural resource management across rural and regional communities. Many LLS staff routinely work in field-based roles at the interface of livestock, wildlife, and environmental systems, making them an important occupational group for understanding zoonotic disease risk and prevention within a One Health context. Other Australian states have similar agencies but with different names. Because LLS staff routinely work at the interface of livestock, wildlife, and environmental systems, they represent an important occupational group for understanding zoonotic disease risk and prevention within a One Health context. This mixed methods study aimed to assess LLS staff knowledge, attitudes, and protective practices regarding JEV to inform One Health–aligned workplace-based prevention strategies.

## 2 Methods

A mixed-methods study was conducted across three LLS regions in NSW: Hunter, Northwest, and Northern Tablelands. These regions encompass coastal, inland, and high-altitude zones in northern NSW with diverse agricultural systems. Eligible participants included LLS staff who undertake field work across animal health, biosecurity, and natural resources. The study adopted a One Health framework to explore occupational risk and cross-sectoral prevention strategies (18).

Data collection occurred in two stages. First, an online questionnaire (see Appendix 1) explored JEV-related knowledge, attitudes, protective behaviours, and perceived information gaps. Second, a focus group (FG) was conducted with consenting staff from one LLS, where preliminary questionnaire findings were presented and explored in greater depth to identify context-specific needs and priorities. Ethics approval was obtained from the University of Newcastle Human Research Ethics Committee (H-2024-0128). Participation in the online questionnaire and FG was voluntary, and informed consent was obtained from all participants prior to data collection.

## 3 Participants and recruitment

The online questionnaire (Microsoft Forms) contained demographic questions and 21 items exploring knowledge, attitudes, and practices using multiple-choice and Likert-scale questions. An invitation email, including study information and a questionnaire link, was distributed to LLS staff by General Managers on behalf of the research team. Up to three reminder emails were sent to staff to encourage participation. Consent was implied through submission of the completed questionnaire. Questionnaire data were collected in September 2024.

Only one focus group (*n* = 20) was conducted, limited to a single LLS region, due to operational and scheduling constraints affecting other participating regions during the study period. The FG was facilitated by an independent qualitative researcher (JW). A FG invitation email, including a participant information sheet was distributed by the by General Managers on behalf of the research team. The FG began with a presentation of the aggregated online anonymised questionnaire findings, followed by discussion of awareness, prevention, and support needs questions (See Table 1).

**Table 1:** Focus Group Guide.

Question	Prompt
• Introductions • Participants asked to describe their role generally and then more specifically potential for exposure to mosquito bites in their work role. • Provide Safe work fact sheet of personal protection practices as a prompt	Expand on proximity to pigs - domestic or feral, and water birds in their work role
• Present results of survey • What are your thoughts or comments after hearing these results?	Expand Are there any surprise findings?
What is your experience of implementing personal protection practices against mosquito bites?	Are there occasions when it is difficult? How do we optimise? Do you lack support in any way? Explore training and access to equipment.
What are your thoughts on vaccination for JEV	Would you get vaccinated? When?
Do you have any suggestions about what could be done to improve the adoption of personal protection practices against mosquito bites?	Explore education and training suggestions. Explore practical personal protection practices needs.
Are there any feedback or suggestions you have going forward?	Explore

## 4 Data Analysis

### 4.1 Quantitative

Questionnaire data were amalgamated before being summarised descriptively using Stata version 17.0 (StataCorp, College Station, Texas, USA). Respondents who did not undertake any field work in their role were excluded. Knowledge, attitudes, and practices were described by frequencies and proportions. Cross-tabulations were prepared to assess differences in attitudes and practices between participants from different LLS regions. Exact univariate odds ratios with 95% confidence intervals were applied.

### 4.2 Qualitative

Focus group data were audio-recorded with recorded participant consent, transcribed verbatim (with identifying data removed) and checked for transcription errors. Two authors (JW, NS), independently coded data using an inductive thematic approach (19). This involved: (i) identifying units of meaning using a process of reading the transcripts line-by-line; (ii) grouping units into categories whereby each category was labelled to assist with retrieval between the data; and (iii) examining relationships between codes in the context of the research question to form themes. To ensure accuracy, consensus coding was undertaken by two researchers (JW, NS). Any differences in researcher perspective were resolved by negotiation and, if necessary, regrouped and recorded until consensus was reached. Regular discussion of interpretations between the wider research team confirmed codes and led to theme development. Credibility and dependability were maintained through consensus coding, reflexive journaling, and peer debriefing (20).

## 5 Results

### 5.1 Quantitative

Fifty-six questionnaire responses were received. Ten respondents were excluded from the analysis as they did not undertake field work. The characteristics of respondents are shown in Table 2. Included respondents were relatively evenly split by gender and mainly university-educated, with long term experience working for LLS. The most common occupations reported by respondents were natural resource management officers (*n* = 9), district veterinarians (*n* = 7), biosecurity officers (*n* = 6), and travelling stock route officers (*n* = 5).

**Table 2:** Local Land Services participant demographics of survey (n = 46) and non-participants (*n* = 10).

Characteristics	Participants (undertake fieldwork) n (%)	Non-participants (do not undertake fieldwork) n (%)
Total	46 (100.0%)	10 (100.0%)
Gender		
Female	21 (45.7%)	10 (100.0%)
Male	24 (52.2%)	0 (0.0%)
Non-binary	1 (2.2%)	0 (0.0%)
Years of experience at LLS		
<1 year	4 (8.7%)	3 (30.0%)
1–2 years	7 (15.2%)	2 (20.0%)
3–5 years	9 (19.6%)	1 (10.0%)
6–10 years	10 (21.7%)	0 (0.0%)
11 years +	16 (34.8%)	4 (40.0%)
Highest level of education attainment		
High school	1 (2.2%)	2 (20.0%)
TAFE/vocational certificate	6 (13.0%)	6 (60.0%)
Bachelor degree	28 (60.9%)	1 (10.0%)
Post-graduate degree	11 (23.9%)	1 (10.0%)
LLS Region		
Hunter	14 (30.4%)	5 (50.0%)
North-West	13 (28.3%)	4 (40.0%)
Northern Tablelands	19 (41.3%)	1 (10.0%)

### 5.2 Knowledge

Most respondents (45/46, 97.8%) had heard of JEV and recognised it as a zoonotic disease, but 76% reported little (19/46, 41.3%) or no knowledge (16/46, 34.8%) of clinical signs. Respondents were generally able to correctly identify mosquitoes, standing water, wearing short sleeved/legged clothing, and pigs as factors affecting JEV persistence and transmission (Table 3). While mosquitoes and pigs were correctly identified as important for the transmission cycle, misconceptions persisted around horses and surface contact-based spread.

**Table 3:** Responses in the baseline survey to knowledge items regarding the role of environmental factors contributing to JEV persistence and transmission.

Environmental factors	Plays a role N (%)	Does not play a role	Not sure	Total respondents
Standing water	39 (86.7%)	4 (8.9%)	2 (4.4%)	45
Long wet grass	23 (52.3%)	12 (27.3%)	9 (20.5%)	44
Water birds	23 (52.3%)	11 (25.0%)	10 (22.7%)	44
Meat	7 (15.9%)	23 (52.3%)	14 (31.8%)	44
Horses	10 (22.7%)	18 (40.9%)	16 (36.4%)	44
Touch	10 (22.7%)	17 (38.6%)	17 (38.6%)	44
Flies	9 (20.9%)	21 (48.8%)	13 (30.2%)	43
Clothing	32 (72.7%)	7 (15.9%)	5 (11.4%)	44
Pigs	31 (70.5%)	5 (11.4%)	8 (18.2%)	44
Mosquitos	42 (91.3%)	2 (4.3%)	2 (4.3%)	46

Note: questions in the baseline survey were not compulsory and respondents could choose to skip questions.

Most participants were uncertain if there were safe work method statements available for mosquito bite prevention in their workplace (35/46, 76.1%).

### 5.3 Attitudes

Participants generally reported concern about contracting arbovirus infections during their field work (Table 4), particularly Ross River virus. Concern regarding JEV infection during fieldwork was mixed. Overall, participants considered the risk of JEV infection during fieldwork as low, but the consequences of an infection to be major. There were no statistically significant differences between the three LLS regions regarding the perceived consequences or likelihood of JEV infection.

**Table 4:** Attitudes of Local Land Services field workers to JEV infection risk.

Attitudes	LLS Region			Total n (%)
	Hunter n (%)	Northern Tablelands n (%)	North-West n (%)	
Concern of JEV infection during fieldwork				
*Yes*	5 (41.7%)	1 (6.7%)	5 (50.0%)	11 (29.7%)
*No*	7 (58.3%)	14 (93.3%)	5 (50.0%)	26 (70.3%)
Concern of RRV infection during fieldwork				
*Yes*	9 (64.3%)	7 (43.7%)	8 (80.0%)	24 (60.0%)
*No*	5 (35.7%)	9 (56.3%)	2 (20.0%)	16 (40.0%)
Concern of BFV infection during fieldwork				
*Yes*	3 (30.0%)	1 (9.1%)	4 (57.1%)	8 (28.6%)
*No*	7 (70.0%)	10 (90.9%)	3 (42.9%)	20 (71.4%)
Perceived likelihood of JEV infection				
*Unlikely*	5 (35.7%)	9 (50.0%)	3 (23.1%)	17 (37.8%)
*Possible*	5 (35.7%)	8 (44.4%)	7 (53.8%)	20 (44.4%)
*Likely*	4 (28.6%)	1 (5.6%)	3 (23.1%)	8 (17.8%)
Perceived consequences of JEV infection				
*Negligible/Minor*	2 (14.3%)	5 (27.8%)	3 (23.1%)	10 (21.7%)
*Moderate*	4 (28.6%)	5 (27.8%)	4 (30.8%)	13 (28.3%)
*Major/Extreme*	8 (57.1%)	9 (50.0%)	6 (46.2%)	23 (50.0%)

Note: questions in the survey were not compulsory and respondents could choose to skip questions. Blank responses were excluded from this analysis.

### 5.4 Practices

PPP use among LLS respondents varied (Table 5). While most participants reported wearing long pants, hats and boots, 76% used mosquito repellent. Notably, 43% reported never reapplying repellent during fieldwork.

**Table 5:** Personal protective equipment practices reported by surveyed LLS staff.

PPE Practices	Hunter n (%)	Northern Tablelands n (%)	North-West n (%)	Total n (%)
Work boots				
*Always*	0 (0.0%)	13 (68.4%)	12 (92.3%)	25 (54.3%)
*Sometimes*	0 (0.0%)	5 (26.3%)	0 (0.0%)	5 (10.9%)
*Never*	14 (100.0%)	1 (5.3%)	1 (7.7%)	16 (34.8%)
Sunscreen				
*Always*	4 (28.6%)	9 (45.0%)	7 (53.8%)	20 (42.6%)
*Sometimes*	10 (71.4%)	10 (50.0%)	5 (38.5%)	25 (53.2%)
*Never*	0 (0.0%)	1 (5.0%)	1 (7.7%)	2 (4.3%)
Hat				
*Always*	4 (30.8%)	7 (36.8%)	7 (58.3%)	18 (40.9%)
*Sometimes*	8 (61.5%)	11 (57.9%)	3 (25.0%)	22 (50.0%)
*Never*	1 (7.7%)	1 (5.3%)	2 (16.7%)	4 (9.1%)
Long pants				
*Always*	12 (85.7%)	17 (89.5%)	11 (84.6%)	40 (87%)
*Sometimes*	2 (14.3%)	2 (10.5%)	2 (15.4%)	6 (13%)
*Never*	0 (0.0%)	0 (0.0%)	0 (0.0%)	0 (0.0%)
Mosquito repellent				
*Always*	0 (0.0%)	2 (10.5%)	1 (7.7%)	3 (6.7%)
*Sometimes*	13 (100%)	11 (57.9%)	10 (76.9%)	34 (75.6%)
*Never*	0 (0.0%)	6 (31.6%)	2 (15.4%)	8 (17.8%)
Mosquito net				
*Always*	0 (0.0%)	0 (0.0%)	0 (0.0%)	0 (0.0%)
*Sometimes*	0 (0.0%)	2 (9.5%)	0 (0.0%)	2 (4.5%)
*Never*	12 (100%)	19 (90.5%)	11 (100%)	42 (95.5%)
I only use whatever mosquito repellent is handy				
*Always*	7 (50.0%)	9 (47.4%)	4 (33.3%)	20 (44.4%)
*Sometimes*	6 (42.9%)	5 (26.3%)	3 (25%)	14 (31.1%)
*Never*	1 (7.1%)	5 (26.3%)	5 (41.7%)	11 (24.4%)
I check the label to see if the mosquito repellent contains picaridin				
*Always*	0 (0%)	1 (5.3%)	0 (0%)	1 (2.2%)
*Sometimes*	1 (7.1%)	1 (5.3%)	1 (7.7%)	3 (6.5%)
*Never*	13 (92.9%)	17 (89.5%)	12 (92.3%)	42 (91.3%)
I check the label to see if the mosquito repellent contains DEET				
*Always*	4 (28.6%)	2 (10.5%)	3 (23.1%)	9 (19.6%)
*Sometimes*	3 (21.4%)	3 (15.8%)	2 (15.4%)	8 (17.4%)
*Never*	7 (50%)	14 (73.7%)	8 (61.5%)	29 (63.0%)
I only use mosquito repellent that contains either picaridin or DEET				
*Always*	0 (0%)	1 (5.3%)	3 (23.1%)	4 (8.7%)
*Sometimes*	12 (85.7%)	11 (57.9%)	3 (23.1%)	26 (56.5%)
*Never*	2 (14.3%)	7 (36.8%)	7 (53.8%)	16 (34.8%)
I reapply mosquito repellent if I am working outside for more the 4 h				
*Always*	4 (28.6%)	2 (10.5%)	1 (7.7%)	7 (15.2%)
*Sometimes*	4 (28.6%)	4 (21.1%)	5 (38.5%)	13 (28.3%)
*Never*	6 (42.9%)	13 (68.4%)	7 (53.8%)	26 (56.5%)
Vaccinated for JEV				
*Yes*	1 (7.7%)	1 (5.6%)	6 (46.1%)	8
*No*	12 (92.3%)	17 (94.4%)	7(53.8%)	36

Note: questions in the surveys were not compulsory and respondents could choose to skip questions. Blank responses were excluded from this analysis.

Eight participants (18.1%) reported a previous JE vaccination. This represents 40% of the LLS respondents who indicated that their workplace provided JE vaccination to field workers. The odds of previous JE vaccination were 12.4 times higher among respondents if the vaccine was offered through their workplace compared to those without workplace-provided vaccination (OR 12.4, *p* = 0.02, 95% CI 1.28–581.7).

### 5.5 Qualitative

A single FG was conducted with one LLS region. The LLS site and information about participants is limited to protect anonymity. Three distinct themes were identified following the FG:

Theme.

1: JE Risk- Awareness and prevention measures.

Theme.

2: Prevention promoted by environmental and temporal cues.

Theme.

3: Benefit of collaboration and cross-sector engagement.

Theme.

1: JE Risk- Awareness and prevention measures.

Participants expressed a strong desire for clear, accessible information about JEV risks and PPP strategies. There was consistent support for wearing long sleeves and trousers as primary prevention. Many participants had accessed PPP information from non-work sources such as television programs. Indeed, participants' comments suggested the need for clarification about mosquito borne disease risk including seasonal risk patterns for JE and confusion about mosquito activity and risk during cooler months.

I am just wondering, do the winter ones pose the same risk for example for the viral transmission or is it a different thing. And like the others have said just to focus on the stereotypical mosquito months during spring and summer (FG, Participant (P) 1)).

Participants raised questions about the safety and effectiveness of different types of repellents (e.g., DEET vs citronella), including which options were most effective and whether they should be applied to the skin, clothing, or both. There was also interest in understanding the effectiveness of repellent-treated clothing and whether washing such clothing affects its protective properties.

It is a serious question - Iike I tend to spray it [repellent] on my clothes now. I try to not put it on my skin much, but you have to if you have exposed areas….are we better to spread on our clothes than on our hands that are sticking out or the feet that are sticking out. (FG, P2).

I know you can get treatment now that you can put on your clothes. So, is there is like a spray that you can put on that is designed specifically not for skin but for clothes. (FG, P3).

Participants reported awareness about vaccination recommendations for outdoor workers. However, not all participants were clear on eligibility and how to access vaccination, whether from GPs or pharmacists. While some participants indicated they had been vaccinated, others expressed vaccine hesitancy, especially following the COVID-19 pandemic. All participants felt that vaccine coordination through the workplace was the best option.

Yeah, there definitely was a push last year for the District Vets [veterinarians] to be vaccinated, which the majority of us are, but …. vaccine hesitancy is an issue because I think following from COVID and that vaccine regime - a lot of people were hesitant to go and get this vaccine. (FG, P4).

Theme.

2: Prevention promoted by environmental and temporal cues.

Participants readily noted they were exposed to mosquitoes in their line of work, and this had the potential to make them complacent. Some participants indicated they were likely to, “roll the dice and think I will be okay” (FG, P8). However, in response to the FG discussion, participants agreed that there was benefit in targeted alerts especially during outbreaks and in peak mosquito seasons.

I always think like, I can handle a few bites, that is not a drama. It would not be on my radar to go out and put on DEET [repellent] every time I went out if there was a chance there might be a mosquito about. But for me I would find it helpful if there was a bit more of a campaign base, where you go, okay from October to March that is a peak time really go hard on it. (FG, P7).

Participants also emphasised the value of visible in-office reminders and field signage and this was considered beneficial for educating about PPP and getting vaccinated during COVID-19. There was also perceived benefit of using field-based events to promote JEV protection and vaccination. Likewise, integrating JE warnings with other zoonotic disease risk messaging was considered important, particularly in communities near piggeries or waterbird populations.

I think posters or something in the workplace as a reminder would be useful. Like we used to have with the COVID posters…..just a reminder to people. Because I know I have a paddock workshop coming up that is not really that far from a piggery, and I have just noticed that that is a risk factor. (FG, P4).

Theme.

3: Benefit of collaboration and cross-sector engagement.

Participants reported that collaboration in zoonotic disease education, such as JE, between multiple organisations such as LLS, health districts, veterinarians and agricultural groups to promote zoonotic disease risk awareness was ad hoc. Participants highlighted the benefit of joint initiatives in the past.

One thing that is missing is what we used to do, I have been with LLS for 10 years now and early in the piece we had staff from the public health unit come and speak at events, field days and those kinds of things. (FG, P11).

That is something that actually the District Veterinarians do for our various districts when we hold events - we give presentations about zoonotic diseases, [such as] Q fever, Leptospirosis, that kind of thing. And … if we are holding events at those peak times, we should note that this is a late afternoon event where mosquitos are going to be more prevalent so, please bring mosquito repellent or dress appropriately. (FG, P4).

There was consensus that integrated communication strategies across all involved agencies to reinforce zoonotic disease messaging would have the greatest impact.

And the gap that we recognised was that vets can only do so much in a human health capacity, and we really needed the partnership with [other organisations]. (FG, P11).

Lengthy handouts were considered less effective, especially for younger people, “to read pages and pages to get [understand] what not to do” (FG, P6). In response the need to incorporate messages into event flyers and leverage social media to reach younger people was noted.

We had these masses of fact sheets that we were giving to people - how many of those 16 or 17-year-olds are going to read eight pages. [We need to have] an easy-to-read brochure, accompanied by a bit of social media posts. (P6).

## 6 Discussion

This study provides timely insights into the knowledge, attitudes, and protective practices of LLS staff, frontline personnel at high risk of JEV exposure through mosquito bites in rural and regional northern NSW. Results from the questionnaire and FG demonstrate inadequate vaccine uptake. We identified the imperative of coordinated vaccination delivery, targeted seasonal messaging, cross-sector collaboration and tailored communication for diverse audiences. The findings highlight both behavioural and systemic challenges to JEV prevention and underscore the importance of embedding these efforts within a One Health framework.

Questionnaire data showed that most participants recognised the value of protective clothing and repellents. However, these measures were inconsistently applied, and misconceptions persisted about transmission and repellent use. These gaps mirror broader challenges in One Health operationalisation, where knowledge alone does not guarantee behavioural change without structural support [21], [22]. Limited clarity around eligibility and access to vaccination further constrained uptake, underscoring the need for streamlined, well-communicated workplace vaccination programs. Embedding vaccination and PPP programs within existing Work Health and Safety systems, alongside clear, seasonal communication, could normalise protective practices and support sustained behavioural change.

Qualitative findings contextualised these gaps. Participants described variable engagement with PPP, influenced by complacency, perceived low risk, or uncertainty about mosquito activity in cooler months. Others reported altering behaviour during peak mosquito seasons, suggesting responsiveness to environmental cues. This variability aligns with previous research linking perceived risk to inconsistent adherence to preventive behaviours [23], [24]. Findings call for clearer, seasonally tailored guidance and stronger workplace leadership to support zoonotic disease prevention.

Although only 18% of respondents were vaccinated, the association between vaccination and workplace provision indicates that access is pivotal. Embedding JEV vaccination within Work Health and Safety frameworks—alongside PPP provision, seasonal alerts, and training—could normalise preventive behaviour and mitigate “vaccine fatigue” following the COVID-19 pandemic (25). These measures would also support organisational preparedness amid increasing zoonotic threats driven by environmental and climatic change.

Cross-sector collaboration emerged as another enabler of One Health action. Participants valued partnerships with Public Health Units and veterinary networks but noted these were often episodic and ad hoc with limited coordination. Instead, a structured, multi-agency approach, linking agricultural biosecurity, environmental management, and human health, was viewed as improving communication consistency, workforce protection, and public awareness. There is existing local evidence that these strong partnerships support early detection of zoonotic threats through shared surveillance data and these should now be used for coordinated messaging (26). Previous One Health initiatives demonstrate that coordinated communication between agricultural, veterinary, and public health agencies can strengthen zoonotic disease preparedness and prevention. For example, integrated vector surveillance and joint risk communication strategies have been successfully used to manage West Nile virus (27) and other mosquito-borne diseases in several settings [28], [29], highlighting the importance of ongoing cross-sector partnerships.

A key insight from the FG was the challenge of communicating zoonotic disease risk to younger staff and community members. Participants felt long fact sheets and brochures were unsuitable, particularly given staff turnover, and preferred concise, visually engaging materials. Consistent with public health evidence (30) participants favoured low-burden strategies such as brief “tag-on” messages in event flyers or digital posts to deliver unobtrusive, integrated communication.

The mixed methods approach, conducted within a One health framework, strengthened the study's conclusions by triangulating quantitative patterns with qualitative insights, providing a nuanced understanding of both behavioural and systemic barriers to JEV prevention. Inclusion of multiple LLS regions supports the transferability of findings across diverse agricultural settings. However, several limitations should be acknowledged. The FG was limited to one region, which may reduce the representativeness of qualitative perspectives. Self-reported data may be subject to recall or social desirability bias, and the modest questionnaire sample size limited the scope for more robust statistical analyses. In addition, concurrent public health messaging about JEV during the study period may have influenced participant awareness and responses.

Future research should evaluate the effectiveness of workplace-based vaccination initiatives and targeted communication strategies emerging from these findings. Longitudinal studies could explore the sustainability of protective behaviours across mosquito seasons and outbreaks. Understanding how zoonotic risk communication can better engage younger or transient agricultural workers also warrants attention.

## 7 Conclusion

Key findings from this study underscore the need for clearer occupational vaccination frameworks, sustained seasonal messaging, and coordinated cross-sector collaboration to strengthen JEV preparedness. Embedding prevention within existing health and safety systems—supported by accessible, audience-appropriate communication—will be key to reducing exposure and improving resilience to future vector-borne disease threats.

J. van der Geer, T. Handgraaf, R.A. Lupton, The art of writing a scientific article, J. Sci. Commun. 163 (2020) 51–59. https://doi-org.ezproxy.newcastle.edu.au/10.1016/j.sc.2020.00372.

## CRediT authorship contribution statement

**Jennifer White:** Writing – review & editing, Writing – original draft, Methodology, Formal analysis, Data curation, Conceptualization. **Nicole Schembri:** Writing – review & editing, Writing – original draft, Methodology, Formal analysis, Data curation, Conceptualization. **Keeley Allen:** Writing – review & editing, Writing – original draft, Methodology, Formal analysis. **Rebecca Forsythe:** Writing – review & editing, Methodology, Conceptualization. **David N. Durrheim:** Writing – review & editing, Writing – original draft, Supervision, Methodology, Funding acquisition, Formal analysis, Conceptualization.

## Consent for publication

Not applicable.

## Ethics approval and consent to participate

Approval for this project was obtained from the University of Newcastle Human Research Ethics Committee (Ref: H-2024-0128). All participants provided written informed consent.

## Funding

The study was conducted with funding from Hunter New England Local Health District, and JW was funded by New South Wales Health through the Prevention Research Support Fellowship.

## Declaration of competing interest

The authors declare there is no conflict of interest.

## Data Availability

Data will be made available on request.

## Appendix A Supplementary data

Supplementary material Image 1

## References

[1] Pearce J.C., Learoyd T.P., Langendorf B.J., Logan J.G. (2018). Japanese encephalitis: the vectors, ecology and potential for expansion. J. Travel Med..

[2] Howard-Jones A.R., Pham D., Jeoffreys N., Eden J.S., Hueston L., Kesson A.M. (2022). Emerging genotype IV Japanese encephalitis virus outbreak in New South Wales, Australia. Viruses.

[3] Moore K.T., Mangan M.J., Linnegar B., Athni T.S., McCallum H.I., Trewin B.J. (2025). Australian vertebrate hosts of Japanese encephalitis virus: a review of the evidence. Trans. R. Soc. Trop. Med. Hyg..

[4] Ladreyt H., Durand B., Dussart P., Chevalier V. (2019). How central is the domestic pig in the epidemiological cycle of Japanese encephalitis virus? A review of scientific evidence and implications for disease control. Viruses.

[5] de Wispelaere M., Desprès P., Choumet V. (2017). European *Aedes albopictus* and *Culex pipiens* are competent vectors for Japanese encephalitis virus. PLoS Negl. Trop. Dis..

[6] OIE Wildlife Health Framework (2020).

[7] de Souza W.M., Weaver S.C. (2024). Effects of climate change and human activities on vector-borne diseases. Nat. Rev. Microbiol..

[8] Pendrey C.G.A., Martin G.E. (2023). Japanese encephalitis clinical update: changing diseases under a changing climate. Aust. J. Gen. Pract..

[9] Sharma R., Agarwal M., Saxena S.K., Kumar S., Gupta M., Mahavar SK S.K. (2025). Japanese Encephalitis: Epidemiology, Pathogenesis, Diagnosis, and Therapeutics.

[10] Misan A., Lambert S.B., Phung H., Young M.K. (2024). Evaluation of the Queensland JEV vaccine program response to the 2022 Australian outbreak. Epidemiol. Infect..

[11] Braddick M., O'Brien H.M., Lim C.K., Feldman R., Bunter C., Neville P. (2023). An integrated public health response to an outbreak of Murray Valley encephalitis virus infection during the 2022–2023 mosquito season in Victoria. Front. Public Health.

[12] Hutchinson D., Kunasekaran M., Chen X., Moa A. (2022). Japanese encephalitis outbreak in South-Eastern Australia, 2022. Glob. Biosecur..

[13] McGuinness S.L., Lau C.L., Leder K. K. (2023). The evolving Japanese encephalitis situation in Australia and implications for travel medicine. J. Travel Med..

[14] Department of Health Disabilty and Ageing National Notifiable Disease Surveillance System. https://nindss.health.gov.au/pbi-dashboard/.

[15] Skinner E., Sartorius B., Furuya-Kanamori L., Craig A., Kiani B., Johnson B B. (2025).

[16] Safe Work: NSW government (2024). Japanese Encephalitis. https://www.safeworkaustralia.gov.au/safety-topic/hazards/japanese-encephalitis.

[17] Ahmad A., Khan M.U., Gogoi L.J., Kalita M., Sikdar A.P., Pandey S S. (2015). Japanese encephalitis in Assam, India: need to increase healthcare workers' understanding to improve health care. PLoS One.

[18] Winkler A.S., Brux C.M., Carabin H., das Neves C.G., Häsler B., Zinsstag J J. (2025). The Lancet One Health Commission: harnessing our interconnectedness for equitable, sustainable, and healthy socioecological systems. Lancet.

[19] Braun V., Clarke V. (2017 May 4). Thematic analysis. J. Posit. Psychol..

[20] Pandit N. (1996). The creation of theory: a recent application of the grounded theory method. Qual. Rep..

[21] Glanz K., Rimer B.K., Viswanath K. (2015).

[22] Potter A., Jardine A., Neville P.J. (2016). A survey of knowledge, attitudes, and practices in relation to mosquitoes and mosquito-borne disease in Western Australia. Front. Public Health.

[23] Schotthoefer A., Stinebaugh K., Martin M., Munoz-Zanzi C. (2020). Tickborne disease awareness and protective practices among US Forest Service employees from the upper Midwest, USA. BMC Public Health.

[24] Oliver J., Larsen S., Stinear T.P., Hoffmann A., Crouch S., Gibney K.B. (2021). Reducing mosquito-borne disease transmission to humans: a systematic review of cluster randomised controlled studies that assess interventions other than non-targeted insecticide. PLoS Negl. Trop. Dis..

[25] Danchin M., Buttery J. (2021). COVID-19 vaccine hesitancy: a unique set of challenges. Intern. Med. J..

[26] Thompson K., Taylor J., Massey P.D., Durrheim D.N. (2024). Members’ experiences and perceptions of participating in an Australian regional one health network. One Health Outlook..

[27] Naveed A., Eertink L.G., Wang D., Li F. (2024). Lessons learned from West Nile virus infection: vaccinations in equines and their implications for one health approaches. Viruses.

[28] Feng X., Jiang N., Zheng J., Zhu Z., Chen J., Duan L. (2024). Advancing knowledge of one health in China: lessons for one health from China's dengue control and prevention programs. Sci. One Health.

[29] Johnson B.J., Brosch D., Christiansen A., Wells E., Well M., Bhandoola A.F. (2018). Neighbors help neighbors control urban mosquitoes. Sci. Rep..

[30] Wakefield M.A., Loken L., Hornik R.C. (2010). Use of mass media campaigns to change health behaviour. Lancet.

