# Supplementary material for: Knowledge, attitudes, and practices of local land services staff regarding Japanese encephalitis virus: A one health perspective
Source: One Health. 2026 May 3;22:101435. doi: 10.1016/j.onehlt.2026.101435 (PMC13158382; doi:10.1016/j.onehlt.2026.101435)
Supplement: Supplementary file 1 — Supplementary material [file mmc1.docx]

Appendix 1: Survey Instrument – Understanding Mosquito-borne Disease in NSW

This appendix presents the survey instrument used to assess knowledge, perceptions, and practices related to mosquito-borne diseases among Local Land Services staff in New South Wales.

SECTION 1: Demographics
1. Gender (Female, Male, Non-binary, Prefer not to say, Other)
2. Years of experience with NSW Local Land Services (<1, 1–2, 3–5, 6–10, 11+)
3. Highest level of education (High school, TAFE, Bachelor’s, Postgraduate)
4. LLS region (Hunter, Northern Tablelands, North-West)
5. Postcode of primary office location

SECTION 2: Work Characteristics
6. Frequency of field work (Likert scale: None -often)
7–8. Field work locations (select from local government area list)
9. Role description (select from list)
10. Field environments (select from list e.g. paddocks, sheds, wetlands)
11. Time of field work (select from list e.g. dawn, day, dusk, night)

SECTION 3: Arbovirus Knowledge
12. Awareness of diseases (select all that apply: JE, MVE, Ross River, Barmah Forest)
13. Awareness of zoonotic transmission (yes, no, not sure)

SECTION 4: Japanese Encephalitis Knowledge
14. Knowledge of clinical signs (Likert scale or knowledge re people, pigs, horses)
15. Perceived likelihood of personal impact (Likert scale)
16. Perceived severity of infection (Likert scale)
17. Perceived role of environmental and animal factors in transmission (Likert scale)

SECTION 5: Risk Perception
18–20. Concern about exposure to JE, Ross River, Barmah Forest viruses (yes, no, not sure)

SECTION 6: Prevention and PPE
21. PPE provided (select from list)
22. Uniform colour (select from list)
23. PPE usage frequency (select from list)
24. Mosquito repellent use behaviours (select from list)
25. Safe Work Method Statement SWMS availability (yes, no, not sure)

SECTION 7: Training and Immunisation
26. Access to SWMS (yes, no, not sure)
27. JE vaccination status (list year)
28–32. Training and information received on mosquito-borne diseases and prevention (yes, no, not sure, provide details)

Note: Full survey wording available upon request or embedded within study materials.
